# A Comparison of High-Impulse and Direct-Current Magnetron Sputtering Processes for the Formation of Effective Bactericidal Oxide Coatings on Polymer Substrates

**DOI:** 10.3390/ma18194591

**Published:** 2025-10-03

**Authors:** Joanna Kacprzyńska-Gołacka, Piotr Wieciński, Bogusława Adamczyk-Cieślak, Sylwia Sowa, Wioletta Barszcz, Monika Łożyńska, Marek Kalbarczyk, Andrzej Krasiński, Halina Garbacz, Jerzy Smolik

**Affiliations:** 1 Łukasiewicz Research Network—Institute for Sustainable Technologies, 26-600 Radom, Poland; 2 Faculty of Chemistry, Warsaw University of Technology, 00-664 Warsaw, Poland; 3 Faculty of Materials Science and Engineering, Warsaw University of Technology, 02-507 Warsaw, Poland; 4 Faculty Environmental Engineering, Warsaw University of Technology, 00-653 Warsaw, Poland; 5 Faculty of Chemical and Process Engineering, Warsaw University of Technology, 00-645 Warsaw, Poland

**Keywords:** silver oxide, copper oxide, magnetron sputtering, HIPIMS, DCMS, bactericidal properties, polymer substrate

## Abstract

In this paper, silver oxide (AgO) and copper oxide (CuO) coatings are placed on a single sputtering target with the direct-current magnetron sputtering (DCMS) and high-power impulse magnetron sputtering (HIPIMS) methods. All the tested coatings are obtained in a reactive process using a metallic target made by the Kurt Lesker company. The investigated coatings are deposited at room temperature on substrates made of pure iron (ARMCO) and polypropylene (PP) without substrate polarization. The deposition time for all the coatings is the same. The results of SEM and TEM investigations clearly show that using the HIPIMS method for the deposition of AgO and CuO coatings reduces their thickness and increases their structure density. Coatings produced with the HIPIMS method are characterized by a higher hardness and Young’s modulus. The value of hardness for AgO and CuO coatings deposited by the HIPIMS method is around 50% higher for AgO coatings and around 24% higher for CuO coatings compared to the coatings obtained by the DC method. This is also true of Young’s modulus values, which are around 30% higher for AgO coatings and 15% higher for CuO coatings produced by the HIPIMS method compared to those of coatings obtained with the DC method. AgO and CuO coatings deposited with both the methods (HIPIMS and DCMS) showed 100% reduction in the viability of two reference laboratory bacteria strains—*Escherichia coli* (Gram−) and *Staphylococcus aureus* (Gram+)—on both types of substrates. Additionally, these coatings are characterized by their hydrophobic properties, which means that they can create a protective barrier, making it difficult for bacteria to stick to the surface, limiting their development and preventing the phenomenon of biofouling. The HIPIMS technology allows for the deposition of coatings with better mechanical properties than those produced with the DCMS method, which means that they are more resistant to brittle fractures and wear and have very good antimicrobial properties.

## 1. Introduction

One of the priorities and challenges of a modern approach to human health in the post-COVID era is to obtain functional materials with antibacterial properties. Daily life requires a new perspective for securing elements in public spaces against the migration of pathogenic microorganisms. Additionally, industries developing on a mass scale require the highest possible adherence to hygiene rules and a new approach to use bactericidal coatings on different components of various machines and devices. Special emphasis has been placed on the use of nanoparticles with antibacterial activities. Currently, inorganic nanoparticles based on metals such as Ag, Au, and Cu, metal oxides like AgO, CuO, TiO_2_, ZnO, and CaO, and ceramic materials with confirmed biocidal properties are widely used [1,2,3,4,5]. Despite numerous and satisfactory achievements in the field of nanotechnology, there are still serious concerns about its use, as the accumulation of nanoparticles (NPs) in the human body may put human health at risk. Many instances of adverse impacts of various metal and metal oxide particles on algae, plants, small invertebrates, fish, and mammal cells have been reported [6,7,8,9,10,11,12,13,14].

To address these problems, new inorganic antiviral materials without nanoparticles but with comparable biocidal properties need to be developed. PVD methods can be one such solution. They can be produced using PVD methods, which will obtain a coating with the properties of nanoparticles, but it will be safer. The correct shape of the microstructure of PVD coatings is crucial not only for their mechanical properties but also for their effective biocidal properties, functionality, and applicability. Therefore, knowledge about the influence of process parameters on the formation of thin-film nanostructures is important. Nanostructure coatings based on silver, zinc, and copper obtained through physical vapor deposition (PVD) are rapidly gaining popularity as core elements of the global strategy to reduce bacterial pathogens [15,16].

One of the methods used to deposit these metals is magnetron sputtering [17,18]. This is a well-known surface-engineering technology and a very effective technological tool enabling deposition on various substrates, including polymer substrates [19,20]. Moreover, it is often used in the process of the deposition of multicomponent coatings. In this case, the authors of the article propose to use two PVD methods to deposit bactericidal oxide coatings on polymer surfaces: DCMS and HIPIMS. The direct-current magnetron sputtering method (DCMS) is well known and traditionally used in the application of functional coatings [21]. High-power impulse magnetron sputtering (HIPIMS), with the use of a pulsed current–voltage supply, is becoming more and more popular. This method uses high-power pulses lasting from a few to hundreds of microseconds [µs] with a low duty cycle of a few percent [22,23]. In some cases, the maximum pulse power may even reach 100–150 kW, which allows the obtaining of average densities of power emitted by the cathode of 1–3 kW/cm^2^. The use of high-power pulses at the target leads to a higher fraction of ionized sputtered material reaching the substrate [24], which in turn enables the deposition of coatings with a dense and fragmented microstructure and excellent adhesion to the substrate [25,26,27,28,29,30,31]. However, it is important to note that while HIPIMS achieves a more ionized flux fraction, it often comes at the cost of a reduced deposition rate compared to traditional sputtering techniques like DC magnetron sputtering. This is because a significant portion of the sputtered ions is attracted back to the target due to the electric field. The HIPIMS method enables changing, giving us the possibility to change the duty cycle used for individual pulses [29], which meets the requirements for lower discharge power. Long intervals between pulses protect the target and substrate from overheating, ensuring process stability. This makes the HIPIMS method the technology that enables an effective deposition of coatings on polymer substrates with a lower thermal resistance as well. An innovative approach is to use polymeric materials as substrates, which is quite common in everyday life.

This article facilitates the understanding of the mechanisms of shaping the microstructure and functional properties of bactericidal oxide coatings by selecting appropriate parameters in the magnetron sputtering processes. In the proposed study, the authors compare the crucial properties of two types of oxide coatings, AgO and CuO, deposited on polypropylene (PP) and pure iron (ARMCO) substrates by means of two methods: DCMS and HIPIMS. All the deposited coatings are analyzed in terms of their structure as well as mechanical and bactericidal properties. The mechanical properties of AgO and CuO coatings, such as hardness and Young’s modulus, are examined on ARMCO with the nanoindentation method using a nanohardness tester by Anton Paar with a Berkovich diamond indenter. The biocidal activity of coatings deposited on a polypropylene (PP) substrate is measured against two reference laboratory microorganisms: *Escherichia coli* (Gram−) and *Staphylococcus aureus* (Gram+) on polypropylene. The wettability of the prepared coatings is also studied by applying the static sessile drop method.

## 2. Materials and Methods

### 2.1. The Coating Deposition Process

**Figure 1:**
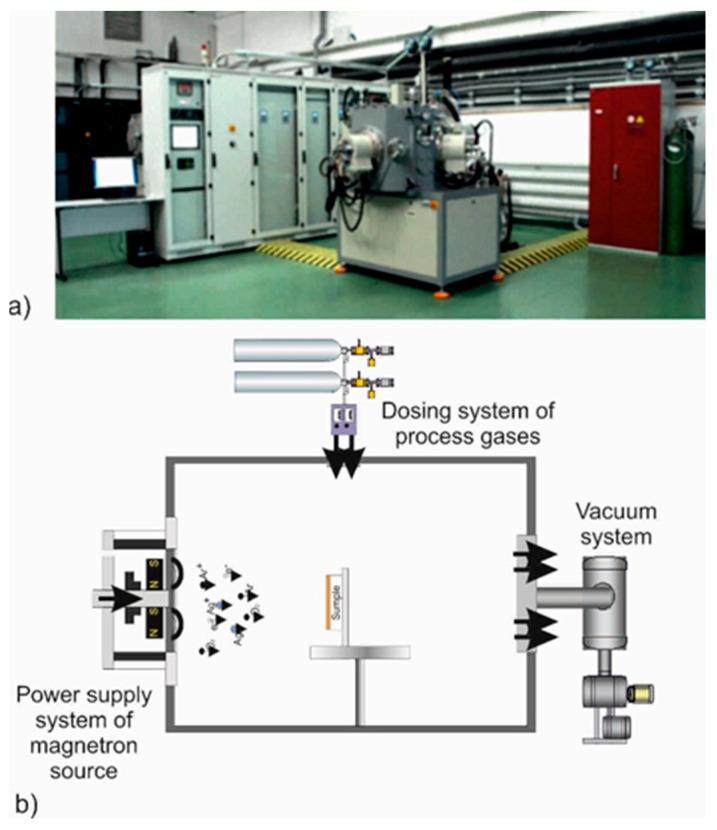
The technological device Standard III (Łukasiewicz–ITEE, Radom, Poland) used for the implementation of plasma surface treatment (**a**) and a scheme of the technological configuration enabling the implementation of the magnetron sputtering processes (**b**).

All studied coatings are deposited on a Standard III technological device (Łukasiewicz–ITEE, Radom, Poland), as shown in Figure 1. For the process of AgO and CuO coating deposition, two targets, Ag (99.99% purity) and Cu (99.99% purity), are used, with a diameter of 1016 mm and a thickness of 7 mm, respectively, produced by the Kurt Lesker company (Jefferson Hills, PA, USA). The distance between the sample and the plasma source (target) is 200 mm. The developed technological configuration is shown in Figure 1b.

The samples are coated at room temperature and in a reactive gas atmosphere with an oxygen and argon mixture. The process of CuO and AgO coating deposition is carried out at constant pressure, with a constant oxygen flow of 30 sccm, and a variable argon flow, adjusted to the thermodynamic conditions of the process, within the range of 300 sccm. An MKS working atmosphere control system (MKS Instruments, Inc., Andover, MA, USA) was installed in the Standard III device (Łukasiewicz–ITEE Radom, Poland), enabling the precise control of gas flows while maintaining a constant pressure in the working chamber. During the coating deposition process, the rotation (2 rpm) of the substrates in the chamber space is introduced. The coatings are produced using average power magnetron sources of 350 W without substrate polarization. The detailed parameters of the deposition process are presented in Table 1.

**Figure 2:**
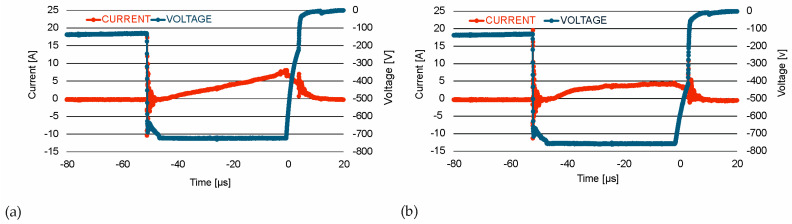
Some fragments of current–voltage curves of powering magnetron sources in the process of AgO (**a**) and CuO (**b**) coating deposition.

In the HIPIMS mode, the target is operated with a pulsing time of 50 μs and a repetition frequency of 1000 Hz. A fragment of the current and voltage curve of powering magnetron sources in the AgO and CuO coating deposition using the HIPIMS method is shown in Figure 2.

The tested coatings are deposited on samples made of pure iron (ARMCO) with a diameter of 25.4 mm, thickness of 6 mm, and surface roughness Ra ≤ 0.03 μm, as well as on samples made of polypropylene (PP) with a diameter of 25.4 mm, thickness of 6 mm, and surface roughness Ra ≤ 0.03 μm. The samples are washed in 99.9% pure alcohol before being placed in the process chamber.

### 2.2. Material Investigation

#### 2.2.1. Microstructure

The surface morphology of the coatings was observed with a Helios 5 PFIB Xe scanning electron microscope (Thermo Fisher Scientific, Hillsboro, OR, USA) with xenon ion sources and using a TOF-SIMS (Time-of-Flight Secondary Ion Mass Spectrometry) detector. This type of surface-analysis technique allows the collection of detailed elemental and molecular information, including chemical imaging and depth profiling. The pulsed ion beam removed molecules from the surface of the studied sample and detected the secondary ions. A secondary electron (SE) detector was used in the observations. TEM sample preparation and STEM observation at 30 kV were performed with the same equipment (Helios 5 PFIB).

#### 2.2.2. Roughness

For microscale roughness measurement, a Form Talysurf PGI 830 profilograph by Taylor Hobson, Warszawa, Poland was used and equipped with a measuring head of 8.0 nm resolution. The measurement employs a laser sensor moving along a tested sample. The maximum number of collected points is 1,600,000. The measuring speed ranges from 0.1 to 2 mm/s. The profilograph has software (MountainMap Universal 7.4.9745) that enables both 2D (profiles) and 3D (topography) analysis, and allows for the determination of roughness and waviness parameters. A roughness value is calculated on a profile (line) and on a surface (area). The arithmetic mean deviation of the roughness profile Ra [µm] and the arithmetic mean deviation of the roughness surface Sa [µm] are measured. For each of the tested surfaces, three measurements were made, and then the average values of these measurements were determined.

#### 2.2.3. Chemical Composition

Energy dispersive X-ray spectroscopy (EDS) was applied to analyze the chemical composition of the coatings with the use of the EDAX Octane Elite Super system (Gatan) (Ametek Inc., Berwyn, PA, USA). The measurements were performed at an acceleration voltage of 5 kV and a beam current of 0.8 nA.

#### 2.2.4. Phase Composition

X-ray diffraction was carried out on a Bruker D8 Advance diffractometer (goniometer radius 280 mm) (Bruker Corporation, Billerica, MA, USA), with parallel beam optics and a Cu Kα radiation (λ = 0.154056 nm) source that operates at 40 kV and 40 mA. The scan optics include a Göbel mirror on the incident beam side and twin secondary Soller slits in the diffracted beam. The measurements are performed with a fixed angle of incidence of the primary beam equal to 2° over the 2θ range between 20° and 100°, with a step size of 0.025° and 3 s counting time per step.

Based on the diffraction results, the Williamson–Hall method was used to estimate the strain, stress, and crystallite size of the samples.

(1) $$\;\frac{\beta \cdot cos\theta }{K\cdot \lambda }=\frac{1}{D_{hkl}}+\varepsilon \cdot \frac{4}{K\cdot \lambda }\cdot sin\theta$$

where:

β is the full width at half maximum,

θ is the Bragg angle,

λ is the wavelength of Cu Kα radiation (1.54056 Å),

K is Scherrer’s constant related to the shape of the crystallites,

D is the crystallite size,

ε is the macrostrain.

The lattice parameters of the individual phases in the investigated samples were determined based on X-ray diffraction data by means of the Nelson–Riley extrapolation method [32].

### 2.3. Mechanical Properties

The pure iron (ARMCO) samples with the deposited coatings are tested for hardness and Young’s modulus, using an Anton Paar Nanoindentation Tester (NHT) (Anton Paar GmbH, Graz, Austria) with a Berkovich diamond indenter. The indentations were made for each of the tested coatings under conditions of load control at two different maximum values of the indenter normal force (1 mN and 2 mN). The loading and unloading rates were 2 mN/min for the intender normal force of 1 mN and 4 mN/min for the intender normal force of 2 mN. The dwell time of the maximum load was 1 s. The indenter penetration depth was measured for each coating and did not exceed 10% of the coating thickness. For each of the tested coatings, a minimum of 10 indentations was made. Correct indentations were selected, and the average hardness (H) and Young’s modulus (E) values were calculated based on the results, as well as the corresponding standard deviations. The measurement results allowed for the determination of the H/E value—the plasticity index or load factor responsible for the maximum elastic deflection when the coating is not destroyed—and the H^3^/E^2^ value—resistance to plastic deformation. The analysis of the mechanical properties allowed for the evaluation of the mechanical resistance of the produced coatings.

### 2.4. Wettability

The pure iron (ARMCO) and polypropylene (PP) samples with the deposited coatings were studied for wettability. For the investigation of the wettability of a plasma-treated surface, a static sessile drop method with demineralized water (drop volume of 2 µL) was used. A goniometer designed and manufactured at Łukasiewicz–ITEE (Radom, Poland) was used. Solid samples covered with all the types of tested coatings were used for the coating wettability measurement. For each sample, 10 measurements of the contact angle were taken, and then the mean value was calculated. Computer image processing improved the speed, accuracy, and precision of the methods used. The Laplace–Young equation [33] for the capillary was solved by the computer software (Analizator v. 1.0.3) in this way:

(2) $$\Delta \rho =\sigma (\frac{1}{R_{1}}+\frac{1}{R_{2}})$$

where:

Δρ is the difference between the density of the drop and the environment;

σ is the interfacial tension;

*R*_1_ and *R*_2_ are the principal radii of curvature.

A digital camera examined the parameters of a drop (diameter, height, etc.), which depended on the angle at which it formed on the surface. The obtained results were compared with the so-called dimensionless (theoretical) profiles, which are solutions to the Laplace–Young equation. The surface or interfacial tension was determined by the following formula:

(3) $$\sigma =\frac{\Delta \rho gR_{o}^{2}}{\beta }$$

where: 

σ is the interfacial tension;

*R_o_* is the radius of the drop at the top;

β is the drop shape parameter;

Δρ is the difference between the density of the drop and the environment;

g is gravity.

The analysis of the contact angle will allow the assessment of the adhesion of contaminants, including bacteria, to the coated surface, which may result in the effect of bacteriostatic activity of the coatings.

### 2.5. Bactericidal Properties

The bactericidal investigation of all pure iron (ARMCO) and polypropylene samples with the deposited coatings was performed using *Escherichia coli* and *Staphylococcus aureus*, a common reference strain in microbiological testing. The tests were repeated three times for each coating. For microbiological analyses, the sterile culture medium Lysogeny Broth (LB), sterile buffer KH_2_PO_4_ (with a concentration of 0.25 mol/L), and sterile laboratory glass (Petri dishes, glass transport vessels, and bottles with closure) were used. The obtained samples were placed on sterile Petri dishes and sterilized under the UV light for 30 min. After 24 h of bacterial culture proliferating, 100 µL of the cell suspension was applied to each sample, and then placed in a glass with approx. 15 cm^3^ sterile buffer KH_2_PO_4_. Next, the samples were covered with aluminum foil and placed in an incubator for 24 h at 37 °C. After incubation, each sample was transferred to a glass bottle containing 9.9 cm^3^ of sterile buffer KH_2_PO_4_ and shaken for 5 min at 180 rpm for the detachment of live bacteria cells from the substrate. In the next step, using the serial dilution method, Petri dishes with the ready culture medium were placed and incubated for 24 h at 37 °C. After incubation, the number of colonies grown on the medium was counted, and the percentage reduction in the viability of *Escherichia coli* (Gram−) and *Staphylococcus aureus* (Gram+) expressed as CFU/mL was calculated using the following formula:

(4) $$R\left[\%\right]=\frac{Iw\;-\;Pp}{Iw}\;\times \;100\%$$

where

Iw is the concentration of bacterial cells in the control [CFU/mL];

Pp is the concentration of bacterial cells in the sample [CFU/mL];

A VHX 6600 digital microscope (Keyence, Mechelen, Belgium) and a 20× objective lens were used for imaging and counting bacterial colonies.

## 3. Results

### 3.1. The Surface Morphology and Microstructure of AgO and CuO Coatings

**Figure 3:**
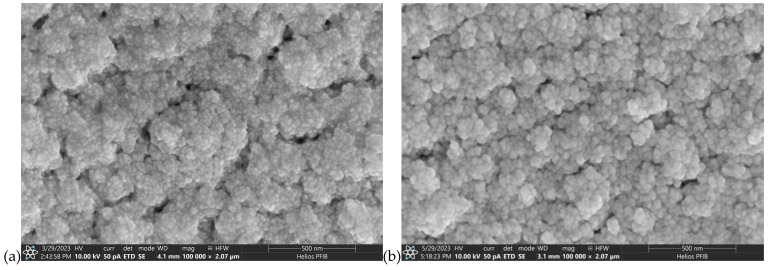

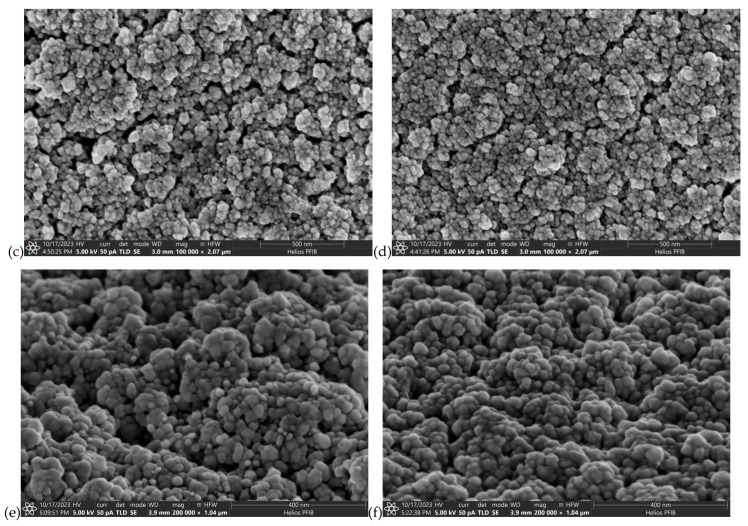
The surface morphology of the AgO coating deposited by different types of magnetron sputtering on pure iron (ARMCO) and polymer substrate: (**a**) DCMS on ARMCO substrate mag. 100,000×, (**b**) HIPIMS on ARMCO substrate mag. 100,000×, (**c**) DCMS on polypropylene (PP) substrate mag. 100,000×, (**d**) DCMS on polypropylene (PP) substrate mag. 100,000×, (**e**) HIPIMS on polypropylene (PP) substrate mag. 200,000×, (**f**) HIPIMS on polypropylene (PP) substrate mag. 200,000×.

**Figure 4:**
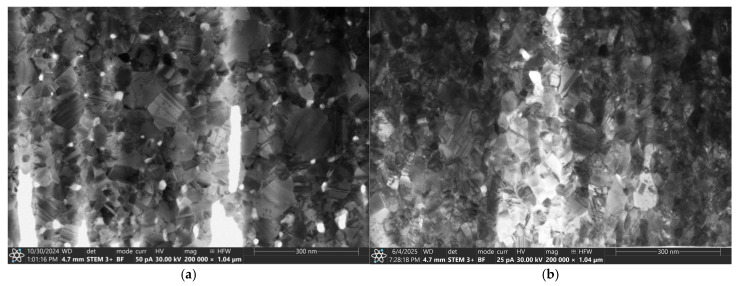
The cross-section of the AgO coating deposited with (**a**) DCMS and (**b**) HIPIMS method.

**Figure 5:**
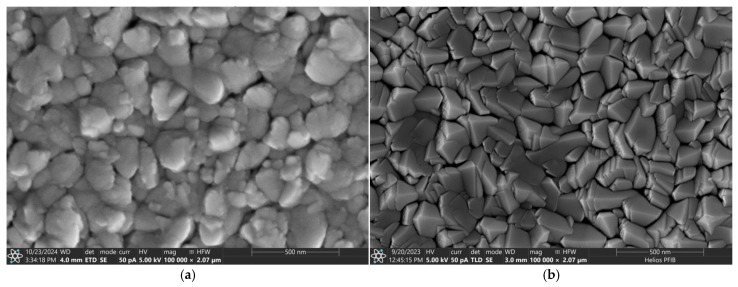

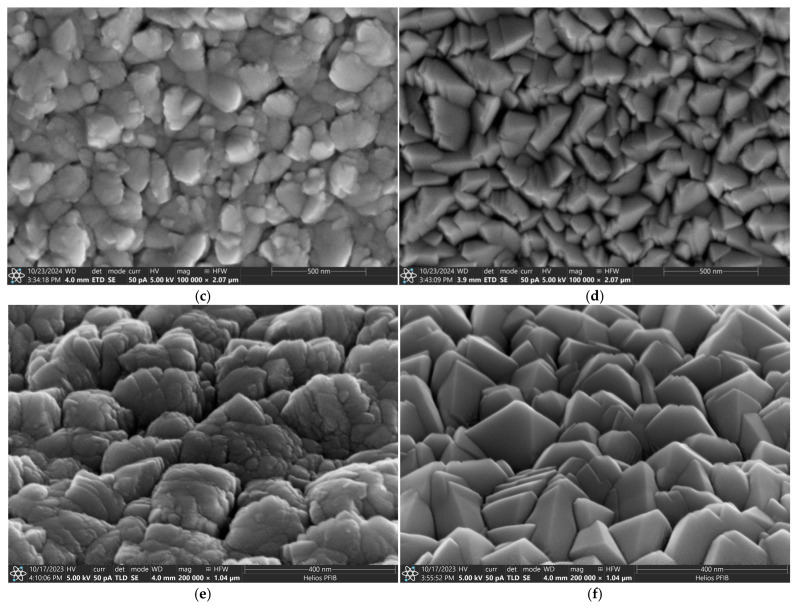
The surface morphology of the CuO coating deposited by different types of magnetron sputtering on pure iron (ARMCO) and polymer substrate (PP): (**a**) DCMS on ARMCO substrate mag. 100,000×, (**b**) HIPIMS on ARMCO substrate mag. 100,000×, (**c**) DCMS on polypropylene (PP) substrate mag. 100,000×, (**e**) DCMS on polypropylene (PP) substrate mag. 200,000×, (**d**) HIPIMS on polypropylene (PP) substrate mag. 100,000×, (**f**) HIPIMS on polypropylene (PP) substrate mag. 200,000×.

**Figure 6:**
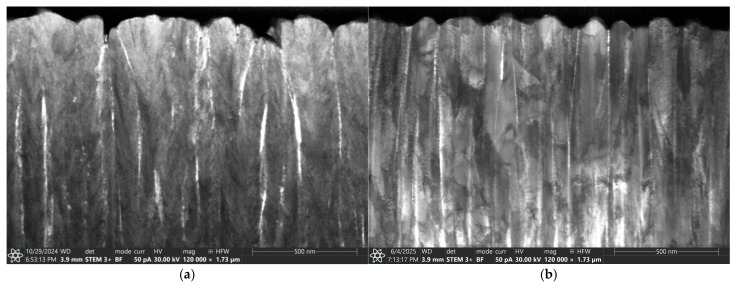
The cross-section of the CuO coating deposited by (**a**) DCMS and (**b**) HIPIMS method.

Some SEM photographs of the surfaces and cross-sections of the AgO and CuO coatings are shown in Figure 3, Figure 4, Figure 5 and Figure 6. SEM observation for AgO_DCMS_ and AgO_HIPIMS_ coatings deposited on pure iron (ARMCO) and polymer substrates shows that they are characterized by a similar morphology in the form of small spherical grains combined into larger agglomerates (Figure 3). The size of particles was in the range of 10 nm to 40 nm, with a larger number of smaller particles observed in the case of AgO_HIPIMS_ coating. The average number of particles per 1 µm^2^ of the surface is 1529 for the HIPIMS method and 1474 for the DC method. In both coatings, small porosities are also visible in the form of spaces between the agglomerates. In the case of coatings obtained using the HIPIMS method, they are much smaller, which indicates that in more compact and homogeneous settings, the coatings are more compact and homogeneous.

The TEM observation of the cross-section (Figure 4) of AgO coatings deposited by the DC and HIPIMS also confirmed the similar structure of these materials. Figure 4a shows a cross-section of the AgO_DCMS_ coating. Its microstructure is formed by equiaxed grains of regular shape. A relatively good contrast is visible due to the difference in crystal orientation. An equiaxial structure is also observed for AgO_HIPIMS_ (Figure 4b) coating, but with a much higher density.

The microscopic observation of the surface of CuO coatings subjected to DCMS and HIPIMS methods on various substrates does not reveal any differences in their morphology. It confirms that the type of substrates does not influence the structure of the coatings (Figure 5). However, the microscopic observation showed significant differences in the morphology of the obtained materials depending on the magnetron sputtering method used. The surface of the CuO_DCMS_ coating shows many irregularly shaped particles resembling agglomerates (Figure 5a,c), while the surface of the CuO_HIPIMS_ coating shows densely packed pyramid-shaped particles characteristic of the CuO phase [34].

There is no variation in particle size. The average particle size was 75.8 nm for the coating obtained with the HIPIMS method and 76.9 nm for the coating obtained with the DC method. The average number of particles per surface of 1 µm^2^ is 111 for the HIPIMS method and 108 for the DCMS method.

The TEM cross-section shows that both types of coatings (HIPIMS and DCMS) are characterized by columnar structure (Figure 6). A more ordered, finer structure with a much higher density is observed in the case of coatings deposited using the HIPIMS method (Figure 6b). This could be attributed to the increased metal ion bombardment commonly seen in HIPIMS discharges.

In Table 2, the chemical composition (in % wt.), thickness, average particle size, and surface roughness Ra/Sa of the CuO and AgO coatings are summarized. The AgO_HIPIMS_ coating (g_AgO-HIPIMS_) is 2000 nm thick and almost three times thinner than the coating produced using the direct-current method (g_AgO-DCMS_ = 5900 nm). The g_CuO-HIPIMS_ coating is 1700 nm thick and two times thinner compared to the coatings produced by DCMS (g_CuO-DCMS_ = 3000 nm). The results confirm a much slower deposition rate of coatings using the high-impulse magnetron sputtering (HIPIMS) method. The often-invoked reason for this is the back-attraction of ionized sputtered material to the target due to a substantial negative potential profile. Recent studies of HIPIMS devices, using floating-emitting and swept-Langmuir probes, show that such extended potential profiles do exist, and the electric fields directed toward the target can be strong enough to seriously reduce ion transport to the substrate [35].

**Figure 7:**
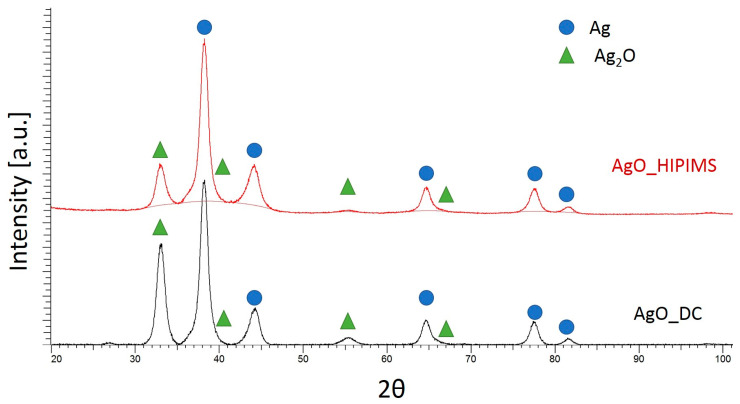
A comparison of the phase structure of AgO coatings deposited with the DCMS and HIPIMS method.

Figure 7 shows the phase analysis of AgO coatings applied by direct-current magnetron sputtering (DCMS) and high-power pulsed magnetron sputtering (HIPIMS). The results show that the coating consists mainly of Ag phase and Ag_2_O regardless of the deposition method. The identified oxide phase differs in its lattice parameter from the standard (PDF card 00–041–1104), which may be related to a different (non-equilibrium) oxide stoichiometry or the high values of residual stresses in the coating formed during its production. A direct comparison of the intensity of diffraction peaks shows that they are higher for the AgO_DCMS_ sample (especially for the Ag_2_O phase). This indicates a higher contribution of this phase compared to the AgO_HIPIMS_ sample. In addition, the diffraction peaks are broad, which may be due to the small size of crystallites in the coating.

**Figure 8:**
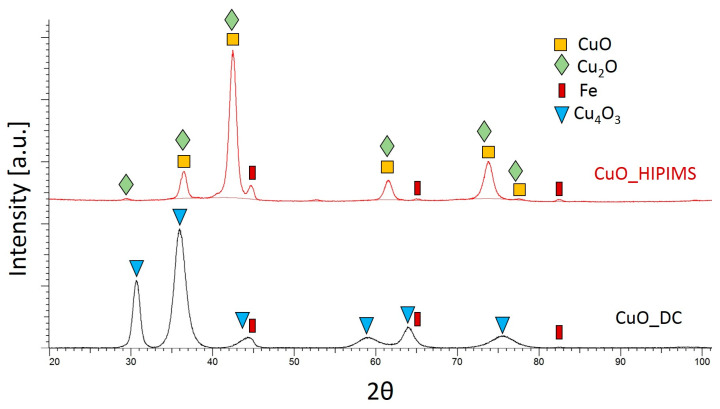
A comparison of the phase structure of CuO coatings deposited with the DCMS and HIPIMS method.

Figure 8 presents diffraction records and the phase analysis of CuO coatings produced with DCMS and HIPIMS techniques. In this case, there is a visible difference in the phase composition depending on the deposition method. In the CuO_HIPIMS_ sample, due to the broadening of the diffraction profiles and similar lattice parameters of the CuO and Cu_2_O phases, the coexistence of these phases cannot be ruled out (the pattern lines of these phases overlap). In turn, for the CuO_DCMS_ sample, the presence of the Cu_4_O_3_ phase is identified. The change in phase structure is most likely related to the increased content of metal ions in the plasma stream used in the HIPIMS method [24]. This greater content produces more copper in the coating, which affects the change in phase composition.

A diffraction peak from Fe (the substrate) is visible in both cases, indicating a smaller coating thickness than AgO. As with the AgO coating, the diffraction peaks are broad, which is responsible for their fine crystallinity.

The lattice parameters are presented in Table 3. In the case of CuO coating produced by the HIPIMS method, the lattice parameters cannot be analyzed because the diffraction peaks of the identified phases overlap, making it impossible to determine the peak broadening in a given phase.

The Ag coating deposited with the DCMS method contains Ag crystallites with a diameter of 5 nm and Ag_2_O with a diameter of 30 nm. The use of the HIPIMS method helps to refine the structure of the obtained coatings by reducing the size of Ag_2_O crystals to 10 nm. The observed differences correlate with the results of cross-sectional observation of these coatings (Figure 4), also indicating a fragmentation of the structure.

### 3.2. The Mechanical Properties of AgO and CuO Coatings

The results of mechanical properties tests show that both the AgO and CuO coatings produced with the DCMS method are characterized by much lower hardness (H_AgO-DCMS_ = 0.8 ± 0.1 GPa, H_CuO-DCMS_ = 3.14 ± 0.2 GPa) compared to AgO and CuO produced using the HIPIMS method. The hardness for HIPIMS coatings is, respectively, H_AgO-HIPIMS_ = 1.4 ± 0.06 GPa, H_CuO-HIPIMS_ = 4.1 ± 0.2 GPa. The hardness of the ARMCO substrates in the form of ARMCO is 1.9 ± 0.06 GPa.

In the case of the AgO coating produced with the HIPIMS method, a significant increase in Young’s modulus (E_AgO-HIPIMS_ = 60 ± 6 GPa) is also observed, compared to the AgO coating deposited with the DCMS method (E_AgO-DCMS_ = 41 ± 4 GPa). The high Young’s modulus for HIPIMS coatings is due to the dense microstructure, consequently enhancing the mechanical properties, which was induced by the dense microstructure and the intense pulse plasma. At the same time, such a high Young’s modulus and hardness mean that the coatings produced with the HIPIMS method are characterized by greater resistance to elastic deformation (H/E_AgO-HIPIMS_ = 0.023, H/E_AgO-DCMS_ = 0.019) and plastic deformation (H^3^/E^2^_AgO-HIPIMS_ = 0.00076, H^3^/E^2^_AgO-DCMS_ = 0.00047). The Young’s modulus of the substrates in the form of ARMCO is 200 GPa.

In the case of the CuO coating, the influence of the deposition technique on the elastic–plastic properties is observed too. The CuO coatings made with the HIPIMS method are characterized by a higher Young’s modulus (E_CuO-DCMS_ = 51 ± 4 GPa, E_CuO-HIPIMS_ = 60 ± 4GPa), higher elastic deformation resistance (H/E_CuO-HIPIMS_ = 0.068, H/E_CuO-DCMS_ = 0.062), and higher resistance to plastic deformation (H^3^/E^2^_CuO-HIPIMS_ = 0.019, H^3^/E^2^_CuO-DCMS_ = 0.012).

### 3.3. Wettability

**Figure 9:**
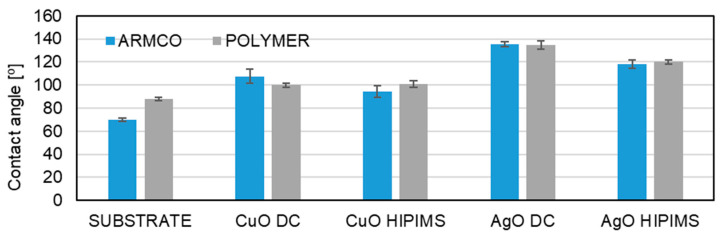
A comparison of the wettability of AgO and CuO coatings deposited using HIPIMS and DCMS.

All coatings have a wetting angle above 90° (Figure 9), which proves the hydrophobic nature of these materials. Hydrophobic surfaces reduce the adhesion of bacteria to a substrate. These materials additionally limit the possibility of bacteria developing and spreading on the surface. The AgO coating deposited using the low-current DCMS method has the highest contact angle. The use of the HIPIMS method resulted in a reduction of the wetting angle to 120°, which is an approximately 20% decrease.

The results for the wettability of the CuO coatings produced with the DCMS and HIPIMS methods (Figure 9) show that these coatings are characterized by similar contact angles oscillating around 100°. Some slight differences are related to measurement errors. There are no differences in the wettability of the coatings depending on the type of substrate.

### 3.4. The Bactericidal Test

**Figure 10:**
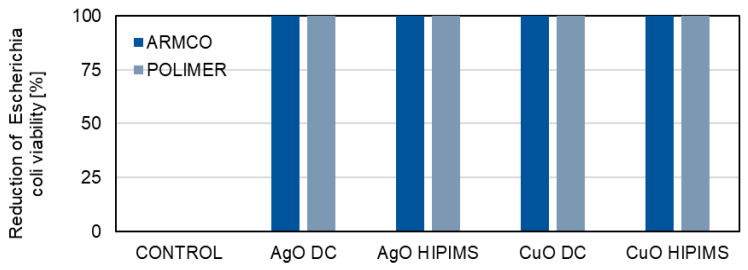
A comparison of the bactericidal properties of AgO and CuO coatings deposited by means of HIPIMS and DCMS against *Escherichia coli* bacteria.

**Figure 11:**
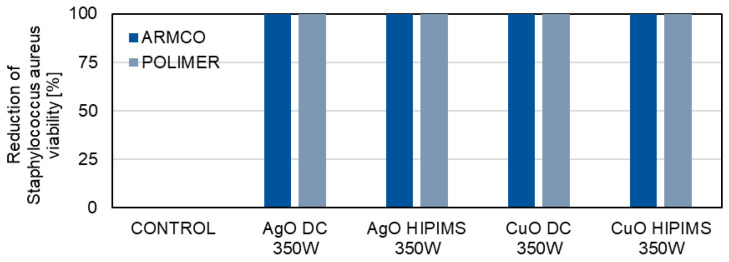
A comparison of the bactericidal properties of AgO and CuO coatings deposited using HIPIMS and DCMS against *Staphylococcus aureus* bacteria.

The microbiological tests are aimed at determining whether the metallic oxide coatings (AgO and CuO) deposited using the DCMS and HIPIMS methods show a similar antibacterial effect (Figure 10 and Figure 11).

The AgO coatings deposited on the ARMCO and polymer substrates with both HIPIMS and DCMS methods have very good bactericidal properties. Similar results are obtained for ARMCO and polymer samples covered with CuO coatings deposited by both the DCMS and HIPIMS methods. For all the tested samples, a 100% reduction in the viability of *Escherichia coli* bacteria (Figure 10) and of *Staphylococcus aureus* bacteria (Figure 11) is achieved, compared to the control sample, for which the reduction of bacterial viability is 0%. The high effectiveness of oxide coatings is related to various mechanisms of the bactericidal characteristics of Ag and Cu [36,37]. Coatings from these metals can exert genotoxic and mutagenic effects, and induce the deformation of cytoplasmic membranes and form reactive oxygen species (ROS), which damage important cellular structures of pathogens.

## 4. Discussion

**Figure 12:**
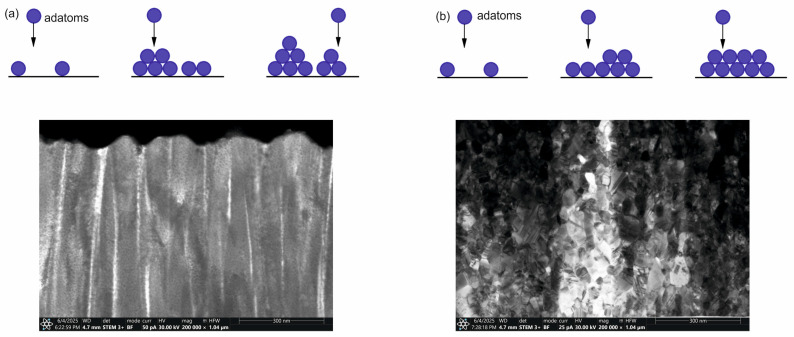
The coating growth mechanism (**a**) island growth mechanism, (**b**) layer-by-layer growth mechanism.

Both types of coatings based on AgO and CuO are generated using two different reactive magnetron sputtering techniques, HIPIMS and DCMS, on two different substrates. The coatings deposited on pure iron (ARMCO) and polymer (PP) have a similar structure, confirming the lack of influence of the substrate on the growth mechanism and surface morphology of the coatings. When comparing the two ensemble groups, including films grown using HIPIMS with their DCMS-grown counterparts, a statistically significant general trend of density increase for HIPIMS films is observed. The structure analysis showed that, depending on the type of element being sprayed (Ag or Cu), there are different coating growth mechanisms. Three basic modes of film growth based on the principal interactions between substrate atoms and deposited atoms [34,38] can be distinguished: (i) Volmer–Weber’s 3D island growth, (ii) Frank van de Merwe’s layer growth (2D growth), and (iii) Stranski–Krastanov’s 3D clusters on top of one or several monolayers. The type of coating growth mechanism depends mainly on the intensity of surface diffusion of adatoms on the substrate, the rate of nucleation, and the rate of crystal growth [39]. As a result of a smaller surface diffusion, adatoms cannot change their position on the surface, contributing to the formation of a material island and, in effect, the local growth of shell fragments. A columnar structure with large grains is created [40]. This phenomenon is characteristic of Volmer–Weber’s 3D island growth and is observed in coatings containing Cu (CuO) (Figure 12a). In the case of greater surface diffusion, atoms can move easily on the surface and nucleate faster. As a result, a dense network of grain embryos is created on a substrate. Additionally, the grain embryos connect, and in this way, the coating grows layer by layer [41,42,43]. Here, the nucleation rate is higher than the crystallization rate. Such a mechanism is observed in coatings containing Ag (AgO) (Figure 12b).

Moreover, a significant impact of the deposition method on the surface morphology and structure of the obtained coatings is demonstrated. AgO coatings produced using the HIPIMS technique are characterized by greater uniformity, density, and structure refinement compared to coatings obtained using the DC method. No influence of the AgO coating deposition technique on their phase structure is observed. Regardless of the deposition method, the AgO coating consists of a mixture of Ag and Ag_2_O phases. The larger share of the Ag phase observed in the coating obtained with the HIPIMS method compared to the one generated by means of the DC method, which also correlates with the results of the chemical composition. The increased silver content is related to the greater intensity of melting of the target material in the HIPIMS method.

In the case of the CuO coating, the use of the HIPIMS method also increased the uniformity of the coatings. Moreover, depending on the deposition method used, changes in the structure of materials are also observed. The CuO coating obtained using the DC technique consists mainly of the Cu_4_O_3_ phase and is composed of grains of irregular shape resembling agglomerates (Figure 5a,c). Using the HIPIMS technique results in a change in the phase structure to a mixture of two phases, CuO and Cu_2_O, which have better antibiofouling properties compared to other types of copper oxide. The densely packed pyramid-shaped grains observed on the surface of CuO_HIPIMS_ coating are characteristic of the CuO phase [34].

The observed changes in the structure of coatings (AgO and CuO), depending on the deposition method, contribute to changes in mechanical properties. Hardness and Young’s modulus are higher for the coatings obtained with the HIPIMS method. In the case of AgO coatings, the value of hardness is around 50% higher, and around 24% higher for CuO coatings, compared to coatings generated with the DC method. A similar situation can be observed for Young’s modulus values. In the case of AgO coatings, the value is around 30% higher and 15% higher for CuO coatings, compared to coatings obtained with the DC method. The observations are consistent with the idea that the density and fineness of the coating increase for HIPIMS and lead to an increase in the hardness of the coating. Coatings produced using the HIPIMS technique are characterized by a higher plasticity index (H/E) and higher resistance to plastic deformation (H^3^/E^2^), confirming their better resistance to brittle fracture. The higher contact angles or amplified wettability observed on the surfaces of rough solid materials are typically expressed as a function of a physical dimension (roughness factor). The roughness can magnify the inherent surface chemistry that seems to have a direct influence on surface wettability. The literature reveals that surface texture and surface energy play significant roles in determining the extent of surface wettability. The contact angles of liquid droplets on solid materials have been employed in the prediction of wettability and surface energies of different materials. Materials with high surface energy are hydrophilic, and water droplets on their surfaces exhibit contact angles below 90°. On the other hand, materials with low surface energy tend to be hydrophobic, where the contact angles of water droplets on their surfaces are greater than 90° [44,45]. Surface wettability tests show that, regardless of the method, coatings have hydrophobic properties. The slight decrease in wettability of coatings deposited using the HIPIMS method is most likely related to the change in microstructure and lower roughness. The microstructure of a coating significantly impacts its wettability through factors like surface roughness, porosity, grain size, texture, phase content, and surface energy [46,47,48,49]. For example, a greater surface roughness can enhance or reduce wettability depending on the liquid and surface properties, and the Wenzel and Cassie–Baxter models describe how liquid interacts with complex microstructures created by roughness. The presence of voids or pores can trap air, leading to hydrophobic surfaces, while a high concentration of reactive phases can form new surface layers that alter the wetting behavior [50,51]. Surface chemistry affects because the type and arrangement of atoms and molecules on a material surface determine interaction (adhesion) forces between the surface and the liquid. Increased adhesion, caused by stronger interfacial interactions, leads to a lower contact angle and better wetting, while weaker interactions result in a higher angle and weaker wetting. Changing the chemical composition of a surface, e.g., by chemical treatment, can increase its hydrophilicity (the surface attracts water molecules) or hydrophobicity (the surface repels water), which is a direct manifestation of a change in wettability [52]. Surface wettability studies also confirm that a thin PVD coating can completely change the character of the coated material. A hydrophilic substrate made of ARMCO after the deposition of AgO and CuO coatings becomes a hydrophobic material (Figure 9). Our study of bactericidal properties showed no influence of the deposition method on the bactericidal properties. Both the coatings obtained using the HIPIMS and DCMS methods display a 100% reduction in Gram+ and Gram− bacteria. Additionally, these coatings are characterized by hydrophobic properties (contact angles with demineralized water above 90°), meaning that they can create a protective barrier, making it difficult for bacteria to stick to the surface, limiting their development and preventing the phenomenon of biofouling.

## 5. Conclusions

This article aims to compare the mechanical and functional properties (wettability and bactericidal properties) of AgO and CuO coatings produced using two methods, i.e., DCMS and HIPIMS. The functional properties of the coatings deposited with both methods are the same, while coatings with better mechanical properties are produced by means of the HIPIMS technology. The HIPIMS method enables a high degree of ionization of deposited material. It leads to the growth of smooth and dense coatings characterized by a homogeneous compact structure and surface development on a nanometric scale. These coatings exhibit a higher hardness compared to those with the DCMS method. The positive effect of the HIPIMS method on the elastic–plastic properties is also observed. Both AgO and CuO coatings deposited using the HIPIMS method are characterized by higher Young’s modulus, higher elastic deformation resistance, and higher resistance to plastic deformation.

No impact of the deposition method on the bactericidal properties of the obtained coatings is observed. Both DCMS and HIPIMS are characterized by a similarly high biocidal activity against *Escherichia coli* (Gram+) and *Staphylococcus aureus* (Gram−) bacteria. CuO and AgO coatings also have hydrophobic properties. It helps to create a protective barrier that makes it difficult for bacteria to stick to the surface, limiting their development and preventing the phenomenon of biofouling.

## Figures and Tables

**Table 1:** The parameters of the deposition process for the analyzed PVD coatings.

Material	Atmosphere [sccm]	Pressure [mbar]	Average Magnetron Source Power [W]	Maximum Current Imax [A]	Maximum Voltage Umax [V]	Temperature [°C]	Time [min]
DCMS							
AgO	Ar:300 O_2_:30	5.0 × 10^–3^	350	0.75	460	20	60
CuO			350	0.75	465		
HIPIMS							
AgO	Ar:300 O_2_:30	5.0 × 10^–3^	350	5	759	20	60
CuO			350	8	752		

**Table 2:** The material parameters of AgO and CuO coatings deposited with direct-current magnetron sputtering methods (DCMS) and high-energy impulse magnetron sputtering (HIPIMS).

Coatings		Thickness [nm]	Deposition Rate [nm/min]	Average Particle Size [nm]	Surface Roughness Ra/Sa [nm]	Cu/Ag Content [%wt.]	O Content [%wt.]
	AgO						
AgO_DCMS_		5900	98.3	20.8	8.29/8.52	93.0	7.0
AgO_HIPIMS_		2000	33.3	20.4	7.11/7.12	95.5	4.5
	CuO						
CuO_DCMS_		3000	50	75.8	3.38/17.6	78.7	21.3
CuO_HIPIMS_		1700	28.3	76.9	3.21/14.6	83.4	16.6

**Table 3:** The lattice parameters of AgO coatings deposited with direct-current magnetron sputtering methods (DCMS) and AgO and CuO coatings deposited with high-energy impulse magnetron sputtering (HIPIMS).

Phase	Crystallite Size [nm]	Microstrain [ε]	Lattice Constant [Å]
AgO DC			
Ag	5	−0.0153	4.0874
Ag_2_O	30	0.0184	4.6769
AgO HIPIMS			
Ag	5	−0.0149	4.0884
Ag_2_O	10	0.0037	4.6685
CuO DC			
Cu_3_O_4_	460	0.0320	4.2516

## Data Availability

The original contributions presented in this study are included in the article. Further inquiries can be directed to the corresponding author.
